# Supplementary material for: Sorting at embryonic boundaries requires high heterotypic interfacial tension
Source: Nat Commun. 2017 Jul 31;8:157. doi: 10.1038/s41467-017-00146-x (PMC5537356; doi:10.1038/s41467-017-00146-x)
Supplement: Supplementary file 2 — Supplementary Software 1 [file 41467_2017_146_MOESM2_ESM.zip › PottsModel/SrcPottsModel/doc/model/Constants.HeatMapParameter.html]

Constants.HeatMapParameter


---


|  |  |  |
| --- | --- | --- |
| **Overview** \| **Package** \| **Class** \| **Use** \| **Tree** \| **Deprecated** \| **Index** \| **Help** | |  |
| **PREV CLASS**   **NEXT CLASS** | **FRAMES**    **NO FRAMES**     **All Classes** |  |
| SUMMARY: NESTED \| ENUM CONSTANTS \| FIELD \| METHOD | DETAIL: ENUM CONSTANTS \| FIELD \| METHOD |  |


---


## model Enum Constants.HeatMapParameter

```
java.lang.Object
  java.lang.Enum<Constants.HeatMapParameter>
      model.Constants.HeatMapParameter
```

**All Implemented Interfaces:**: java.io.Serializable, java.lang.Comparable<Constants.HeatMapParameter>

**Enclosing class:**: Constants

---

``` public static enum Constants.HeatMapParameter extends java.lang.Enum<Constants.HeatMapParameter> ```

---

**Enum Constant Summary**

`ANGLE`

`ANGLE_ENERGY`

`ENERGY`


| **Method Summary** | |
| --- | --- |
| `static Constants.HeatMapParameter` | `valueOf(java.lang.String name)`             Returns the enum constant of this type with the specified name. |
| `static Constants.HeatMapParameter[]` | `values()`             Returns an array containing the constants of this enum type, in the order they are declared. |

**Methods inherited from class java.lang.Enum**

`clone, compareTo, equals, finalize, getDeclaringClass, hashCode, name, ordinal, toString, valueOf`

**Methods inherited from class java.lang.Object**

`getClass, notify, notifyAll, wait, wait, wait`

**Enum Constant Detail**

### ANGLE

```
public static final Constants.HeatMapParameter ANGLE
```

---


### ANGLE\_ENERGY

```
public static final Constants.HeatMapParameter ANGLE_ENERGY
```

---


### ENERGY

```
public static final Constants.HeatMapParameter ENERGY
```


**Method Detail**

### values

```
public static Constants.HeatMapParameter[] values()
```

:   Returns an array containing the constants of this enum type, in
    the order they are declared. This method may be used to iterate
    over the constants as follows:

    ```
    for (Constants.HeatMapParameter c : Constants.HeatMapParameter.values())
        System.out.println(c);
    ```

    :   **Returns:**: an array containing the constants of this enum type, in the order they are declared

---


### valueOf

```
public static Constants.HeatMapParameter valueOf(java.lang.String name)
```

:   Returns the enum constant of this type with the specified name.
    The string must match *exactly* an identifier used to declare an
    enum constant in this type. (Extraneous whitespace characters are
    not permitted.)

    :   **Parameters:**: `name` - the name of the enum constant to be returned. **Returns:**: the enum constant with the specified name **Throws:**: `java.lang.IllegalArgumentException` - if this enum type has no constant with the specified name: `java.lang.NullPointerException` - if the argument is null


---


|  |  |  |
| --- | --- | --- |
| **Overview** \| **Package** \| **Class** \| **Use** \| **Tree** \| **Deprecated** \| **Index** \| **Help** | |  |
| **PREV CLASS**   **NEXT CLASS** | **FRAMES**    **NO FRAMES**     **All Classes** |  |
| SUMMARY: NESTED \| ENUM CONSTANTS \| FIELD \| METHOD | DETAIL: ENUM CONSTANTS \| FIELD \| METHOD |  |


---
